# Prevalence and Associated Factors of Codependency Among Wives of Alcohol Users

**DOI:** 10.1002/puh2.70231

**Published:** 2026-07-22

**Authors:** Savitha Prabhu, Binil Velayudhan, Prachi Pundir, Olga Gershuni

**Affiliations:** ^1^ Department of Psychiatric (Mental Health) Nursing Manipal College of Nursing Manipal Academy of Higher Education Manipal India; ^2^ Co‐Founder and Co‐CEO Collaboration for Evidence Synthesis Navelim Goa India; ^3^ Research Fellow The George Institute for Global Health New Delhi India; ^4^ International Faculty Manipal College of Nursing Manipal Academy of Higher Education Manipal India; ^5^ Department of International Health Care and Public Health Research Institute—CAPHRI, FHML Maastricht University Maastricht the Netherlands

## Abstract

**Background:**

Codependency refers to an excessive emotional or psychological dependence on a partner, typically one who is struggling with addiction or illness and requires continuous care. Among wives of alcohol users, codependent behaviors are commonly observed and can significantly impact their mental health, social functioning, and overall well‐being. Despite the growing recognition of this issue, there is a lack of comprehensive evidence summarizing the prevalence and associated factors of codependency in this population. This scoping review aims to address this gap in the existing literature.

**Methods:**

A comprehensive search was conducted in PubMed (MEDLINE), CINAHL, Scopus, Web of Science, and Embase for studies published in English since 2000. Records were screened independently by two reviewers using predefined eligibility criteria. Data were extracted using the Joanna Briggs Institute (JBI) tool and summarized narratively and in tabular form.

**Results:**

Eighteen studies involving 2299 participants from India, Iran, Pakistan, and Turkey were included. Thirteen studies reported prevalence rates of codependency among wives of alcohol users, ranging from 25% to 98%, with most indicating mild to moderate levels. Three major domains were identified as associated factors: (1) psychological (depression, anxiety, and low self‐esteem), (2) social (marital life, family interaction, interpersonal relationship, social support, and coping), and (3) physical (trauma or abuse history).

**Conclusion:**

This scoping review highlights that codependency is a common and multifaceted issue among wives of alcohol users. The findings can inform the development of targeted interventions and policies aimed at promoting the mental health and overall well‐being of spouses of alcohol users.

## Graphical abstract

This scoping review synthesizes evidence on the prevalence and associated factors of codependency among wives of alcohol users. Findings from 18 studies reveal high rates of codependency (25%–98%), influenced by psychological, social, and physical factors, highlighting the need for targeted psychosocial interventions.

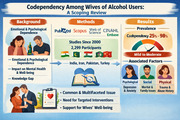

## 1 Introduction

The term “codependency” was originally used to describe behaviors observed in relationships involving individuals with substance use problems. It refers to excessive emotional or psychological dependence on a partner, often one who is addicted, unwell, or in need of care. Codependency may develop because of early life neglect, dysfunctional family dynamics, or exposure to trauma [1]. It encompasses a range of maladaptive behaviors and dysfunctional thinking patterns commonly exhibited by parents, spouses, or children who are strongly associated with a person having an alcohol or drug use disorder (AUD) or dependence on other harmful substances. These patterns often lead to significant psychological and emotional distress among affected family members [2].

Alcohol dependence syndrome (ADS) is both a social and familial disorder, as its roots often lie within dysfunctional family dynamics, and it profoundly affects marital relationships [3]. Addiction impacts not only the individual but also the entire family, as relatives may intentionally or unintentionally support, enable, or shield the substance user [4]. Problematic alcohol use has detrimental effects on the spouse, who may experience physical and mental health issues, fatigue, self‐pity, resentment, and social withdrawal. The spouse often assumes dual parental roles, bearing the responsibilities of both partners. In this process, the nonalcoholic partner may become emotionally strained, erratic, or demanding and may at times neglect the children to hold the family together [4, 5].

Wives of alcohol users often exhibit codependent behaviors that negatively affect their mental health, social functioning, and overall well‐being. They tend to overextend themselves to meet others’ needs, avoid conflict, and comply with unreasonable demands to maintain acceptance and self‐worth [4, 6]. Research indicates that spouses of individuals with ADS demonstrate significantly higher levels of codependency compared to healthy controls [3]. Common manifestations of codependency include denial, enabling behaviors, low self‐esteem, over‐identification with the partner, and a diminished sense of spiritual connection [7].

A cross‐sectional study of 845 young women in Mexico City found a 25% prevalence of codependency significantly associated with submissive gender roles, histories of abuse, and partners’ alcohol dependence [8]. In Pakistan, codependency correlated positively with age, duration of marriage, and a spouse's substance use [6]. Indian studies reported moderate codependency in 56%–72% of wives of men with alcohol dependence [9, 10]. Conversely, a US survey found low codependency levels among 99% of participants, though depression was strongly linked to codependent traits [11].

Despite experiencing severe social and emotional difficulties, codependent individuals often engage in problematic relationships, typically with chemically dependent partners, and continue to provide care and support for them. Components of interpersonal fulfillment, such as stress management, communication, conflict resolution, intimacy, emotional regulation, and personal boundaries, as well as interpersonal satisfaction, are often compromised in these relationships [12, 13]. Husbands who misuse alcohol commonly face marital problems, leading to frequent separations or divorces due to their unpredictable behavior, lack of understanding, and neglect of family responsibilities [14, 15]. Alcohol misuse further exacerbates marital distress, as couples in which one or both partners misuse alcohol report higher levels of marital conflict compared to couples without alcohol‐related problems [16]. One study also found that greater codependency was associated with more troubled personal relationships, with both partners perceiving each other's behavior more negatively in stressful situations [17].

Research consistently shows that codependency hurts relationship quality. It is inversely associated with interpersonal locus of control [18], marital quality [12], interpersonal satisfaction [13], social functioning, and happiness, whereas it is positively associated with marital stress [17, 19]. Low self‐confidence has been identified as a strong predictor of codependency [20]. Codependent traits also correlate positively with dependent and borderline personality features [21]. Support groups in the United Kingdom further highlight common codependent patterns, including a poor sense of self, emotional and relational imbalance, and unresolved childhood issues [22]. Among women, codependency is intricately linked to depression, low self‐esteem, unresolved family‐of‐origin conflicts, and health problems [23]. Indian studies report that wives of men with AUDs experience significantly higher depression, anxiety, and marital distress compared to wives of non‐AUD men [24, 25].

The idea of codependency is still controversial and poorly defined, despite its extensive application in clinical and research settings. Codependency has been conceptualized as a maladaptive coping style, a relational pattern marked by excessive emotional or behavioral reliance on a partner, or a personality‐related vulnerability. It originated from addiction treatment and self‐help movements [11, 26]. Codependency was operationalized using a variety of thresholds and measurement methods across the studies included in this evaluation, resulting in variations in reported prevalence and related characteristics. Academics have challenged the notion for lacking conceptual clarity and for perhaps pathologizing caregiving activities, especially in women, without taking cultural and environmental norms into sufficient consideration [27, 28]. In collectivist and patriarchal cultural contexts, where self‐sacrifice, perseverance, and marital commitment are socially reinforced, this issue is particularly pertinent. Additionally, behaviors labeled as codependent may not be psychopathology but rather culturally normative role expectations [7, 10]. Therefore, rather than being a distinct clinical entity, codependency may be best viewed as a multifaceted construct shaped by psychological distress, relational power dynamics, and sociocultural context. Interpreting prevalence numbers and related parameters provided in this review requires an understanding of these conceptual disputes. Given the substantial psychological and relational burden of codependency, addressing this issue is essential. This scoping review, therefore, aims to synthesize existing evidence on codependency among wives of alcohol users and identify research gaps to guide future interventions.

This review specifically examines wives of individuals with AUD, aligning with the focus of existing research and the sociocultural contexts where most studies took place. In many low‐ and middle‐income countries, especially in South and Southeast Asian settings, wives are often the main caregivers and are disproportionately impacted by the psychosocial, economic, and relational effects of their partner's alcohol consumption [10, 29]. Gendered marital roles, financial reliance, and societal expectations of endurance and caregiving increase wives’ risk of developing codependent behaviors [7]. Although other family members and partners can face similar challenges, most empirical evidence has focused on wives, leading to limited data on husbands, male partners, or other caregivers. Therefore, this review was intentionally limited to wives to ensure a clear conceptual understanding and meaningful synthesis of the findings, though this choice may restrict the applicability of the results to other partner groups.

## 2 Methods

This scoping review was conducted in accordance with the Joanna Briggs Institute (JBI) guidelines for scoping reviews [30], and the PRISMA‐ScR checklist was used for transparent reporting [31]. Additionally, the framework proposed by Arksey and O'Malley [32] was adopted to guide the review process.

### 2.1 Stages of Scoping Review

This framework outlines six distinct stages that constitute the scoping review methodology:

#### 2.1.1 Stage 1: Identifying the Research Question

- **Primary**: What is the prevalence of codependency among wives of alcohol users?
- **Secondary**: What factors are associated with codependency in this population?

To align with the research problem, this scoping review employed the PCC framework for prevalence studies (Population, Concept, and Context) guided by the selection of eligibility criteria (Table 1).

**TABLE 1:** PCC framework for eligibility of studies.

Elements of PCC	Inclusion criteria	Exclusion criteria
Population	Wives of alcohol users cohabit with partners irrespective of age, employment status, or living arrangement	Wives of other substance users Studies focusing on non spousal relationships
Concept	Level/degree of codependency among wives of alcohol users Codependency and its associated factors (psychological, physical, and social factors)	—
Context	No restriction on the region (the scoping review included studies across the world) or the setting (community, hospital, and outreach)	—
Types of evidence sources	Qualitative, quantitative, and mixed‐method studies, as well as reviews and grey literature, are relevant to the research question	Studies do not explicitly address codependency

#### 2.1.2 Stage 2: Identifying Relevant Studies

A comprehensive search strategy was employed using a combination of search terms covering all possible variations related to *codependency* and *partners or spouses of alcohol users*. The databases searched included PubMed (MEDLINE), CINAHL (EBSCO), Scopus (Elsevier), Web of Science (Clarivate), and Embase (OVID). The search was conducted by combining concepts and keywords using both controlled vocabulary and free‐text terms along with appropriate Boolean operators. A grey literature search was also performed on the first 10 pages of Google Scholar using keywords such as *codependency, wives of alcoholics, alcohol users, mental health, associated factors*, and *predictors*. In addition, preprint repositories, such as *medRxiv* and *arXiv*, were searched for relevant studies in progress or awaiting publication. Only research studies published in English within the past 25 years (from 2000 onwards) were included in the review.

#### 2.1.3 Stage 3: Study Selection

##### 2.1.3.1 Screening Process

The search results were combined and deduplicated using the Rayyan web application [33]. The unique records were then subjected to two stages of screening. Title and abstract screening, followed by full‐text screening, were conducted independently by two reviewers (BV and SP) based on predefined inclusion and exclusion criteria. Any discrepancies between the reviewers were resolved through discussion with a third reviewer (PP). This scoping review considered empirical studies examining the prevalence of codependency and its associated factors among wives of individuals with AUD. Although review articles and grey literature were initially screened to map the breadth of existing evidence and to identify relevant primary studies, only observational primary studies (cross‐sectional, descriptive, and correlational designs) reporting original data were included in the final synthesis. Reviews, commentaries, and grey literature sources that did not present primary prevalence data were excluded from the results to ensure consistency and comparability of findings.

#### 2.1.4 Inclusion Criteria

- Studies focusing on the codependency of wives of alcohol users
- Studies addressing codependency‐related prevalence and factors associated with codependency
- Peer‐reviewed primary research studies using qualitative, quantitative, or mixed methods
- Peer‐reviewed articles published at any time and written in the English language.

#### 2.1.5 Exclusion Criteria

- Studies focusing on non spousal relationships.
- Studies focusing on the codependency of wives of individuals using other psychoactive substances.
- Studies do not explicitly address codependency.

#### 2.1.6 Stage 4: Data Extraction and Charting

The JBI standardized data extraction tool was used to extract relevant data from the included studies. All three reviewers initially piloted the data extraction form on at least four research studies, after which necessary modifications were made to refine the extraction process. The final data extraction was carried out using Microsoft Excel (Table 2).

**TABLE 2:** Data extraction.

Sl. no.	Authors, country, and year of publication	Aim of the study	Study design, sample, and sampling technique	Assessment tools used	Prevalence of codependency	Characteristics of spouses of alcohol users
1.	(Kaur 2016), India	To assess the depression and codependency among the wives of alcoholics in community settings	Descriptive survey 212 wives of alcoholics Total enumerative sampling technique	Modified Zung self‐rating depression scale Modified Span–Fisher codependency assessment scale	Overall, 72.2% Wives of alcoholics had a medium level of codependency	Most wives of alcoholics (28.3%) were in the age group of 25–29 years, and 34.0% of them had a high school education. Overall, 42.9% were homemakers, and (61.8%) were from nuclear families. Overall, 37.7% had two children
2.	(Paul et al. 2018), India	Assess the codependency and quality of marital life among spouses of patients with alcohol dependence (ADS)	Descriptive survey 80 spouses of patients with ADS were selected using a convenient sampling technique	Socio‐personal proforma Span–Fischer Codependency scale ENRICH Quality of Marital Life Scale	The majority (48.75%) of the subjects were moderately codependent, 41.25% of the subjects were severely codependent, and only 10% had mild codependency	The majority (33.75%) of the samples belonged to the 46–65‐year age group, and 75% of the participants belonged to a nuclear family. Most of the subjects, 52.5% followed the Hindu religion, 32% of the subjects had primary education, 43.75% of them were unemployed, and 47.5% of the subjects had a monthly income between Rs. 5000 and Rs. 15,000, and 52.5% had a duration of marital life of more than 20 years
3.	(Sreejamol et al. 2022, India	To assess the codependency and depressive symptoms of wives of alcoholics	Descriptive study 100 wives of alcoholics Sampling technique: convenience sampling technique	Spann–Fischer codependency assessment tool Beck's Depression Inventory	Overall, 63% of the wives had severe codependency, 3% had mild codependency, and 34% had a moderate level of codependency	Most (70%) of the subjects belong to the age group of 35–45 years, and 62% of them belong to the Hindu religion, and 80% had a high school education
4.	(Salonia et al. 2021), India	To assess the codependency and coping strategies among spouses of substance abusers	Descriptive study 100 spouses of patients with 50 opioid and 50 alcohol dependence syndrome	Sociodemographic proforma Spann–Fischer codependency scale Coping questionnaire Proforma for severity of addiction	All the spouses showed codependency, with 60% of the spouses showing higher codependency	The majority, 78%, of them were spouses of alcoholics. Overall, 56% of the spouses of alcoholics studied up to matriculation. Overall, 2% of the spouses of alcoholics were not aware of their husband's substance abuse problem, and the type of substance abused
5.	(Nandhini 2021), India	To assess the correlation between codependency and the quality of marital life among wives of alcohol‐dependent clients	Descriptive correlational design 50 wives of clients with alcohol dependence A non‐probability purposive sampling method	Sociodemographic proforma Codependency scale Marital quality scale	The majority, 92% of respondents, have a high level of codependency, and the remaining 8% of them have a moderate level of codependency	Most (40%) of the wives of alcoholics belong to the age group between 30 and 35 years, and 46% of their wives had a high school education. Overall, 82% of wives were homemakers. The majority, 70% of them, belong to the nuclear family. Most of them (48%) had 11–20 years of married life
6.	(Rajamani and Jyoti 2021), India	The study aims to assess the codependency and quality of marital life among spouses of patients with alcohol dependence syndrome (ADS)	Descriptive survey 60 spouses of patients with ADS A convenient sampling technique was used	Socio‐personal proforma Span–Fischer codependence scale ENRICH Quality of Marital Life Scale	Overall, 60% of subjects had an average level of codependency, with the mean score of codependency level being 49.78, and the standard deviation value was 17.41	No specific information
7.	(Senthil 2016), India	To compare the family interaction and codependency in spouses of alcohol dependence in comparison with normal controls	Cross‐sectional study 60 spouses of individuals with alcohol dependence (30 with alcohol dependence and 30 spouses of normal individuals) Purposive sampling technique	1.Alcohol dependence questionnaire General health questionnaire Sociodemographic data sheet Family interaction pattern scale Codependency scale	The level of codependency was significantly higher in the spouses of individuals with ADS (55.60 ± 12.62) as compared to the spouses of normal individuals (47.03 ± 9.49)	The mean age of spouses of alcohol‐dependent patients was 36.46 ± 7.33. Spouses of ADS patients were significantly less educated (*p* < 0.05) compared to normal controls. Most of the spouses of ADS patients were from lower socio‐economic status, nuclear families, and housewives and belonged to the Hindu religion
8.	(Sarkar et al. 2015), India	To assess codependence in spouses of substance‐dependent men in a developing country and to evaluate its relationship with other clinical factors	Descriptive survey A total of 100 spouses of alcohol‐ or opioid‐dependent men seeking treatment at a de‐addiction center in India were recruited	Sociodemographic and clinical data Codependency assessment questionnaire	Co‐dependence was present in 64% and 56% of the spouses in alcohol and opioid groups, respectively	The mean age of the spouses of alcohol dependents was 38.7 ± 8.5. The mean duration of marriage is 17 ± 9.8
9.	(Baby et al. 2024), India	To assess the codependency and personality traits of caregivers of ADS patients	A cross‐sectional study 92 male ADS patients’ caregivers were evaluated	The International Personality Disorder Examination (IPDE) scale Spann–Fischer codependency scale (SFCS)	Most of the caregivers, 57.61% had low, 29.35% had moderate, and 13.04% had severe codependency Among 67 wives of patients, 41 (61.2%) had low codependent scores	Among the 92 caregivers, 67 (72.8%) were wives of the patients
10.	(Devi et al. 2020), India	To assess the codependency and depressive symptoms among wives of alcoholics	Descriptive study 100 wives of alcoholics convenient sampling	Background proforma Codependency scale Centre for epidemiological studies depression scale	55% of the sample was found to be moderately codependent scoring 16–39, and 45% was found to be highly codependent	Most of the wives, 34%, were between 26 and 30 years of age. A significant number of wives, 28% were in consanguineous marriages. The duration of stay was more than 10 years in 58% of the participants. About 40% of the wives had no formal education. Regarding occupation, 45% were housewives, 47% were going for daily wages, and 8% were self‐employed
11.	(Zaidi 2015), Lahore, Pakistan	To assess the codependency and relationship satisfaction among spouses of alcohol abusers	Correlational study 70 female spouses of an alcohol abuser	Sociodemographic information form Am I a codependent scale Relationship satisfaction survey	Not assessed	Most of the spouses of alcohol abusers are aged between 26 and 30 years. Most of the participants have done intermediate schooling (42.9%). The majority (68.6%) were living in a nuclear family structure
12.	(Sawane et al. 2014), India	To assess the level of codependency and depressive symptoms among wives of alcoholics	A descriptive correlational design 100 wives of alcoholics Non‐probability convenience sampling technique	Sociodemographic proforma Modified Span–Fischer codependency scale Centre for epidemiologic studies depression scale (CES‐D)	52% of the subjects were found to be moderately codependent, and 48% were found to be highly codependent	Most (48%) of subjects were in the age group of 20–30 years. Regarding the number of children, 26% had only one child, 34% had two children, 32% had three children, and only 8% of the sample had four children. Many (45%) of the sample had no formal education, 30% had primary education, and 25% had secondary education. About 42% of subjects were housewives, 40% were daily wagers, and 18% were self‐employed. The majority, 92%, were Hindus, and 8% were Muslims. About 80% of the samples belonged to a nuclear family
13.	(Bhowmick et al. 2001), India	To examine the relationship between social support, coping resources, and codependence in the wives of individuals with drug and alcohol dependence	A descriptive correlational design 60 wives (30 each of alcohol and drug dependence), husbands	Social support scale (SSS) Coping resources inventory (CRI) codependence assessment questionnaire (CAQ)	Of the sixty patients, 49 (81.66%) were found to be codependent Out of 49 codependent wives, 27 (55%) were from the drug group and 22 (44.9%) were from the alcohol group	Most of the wives were in the age group of 20–30 years (mean age for drug group = 31 years; mean age for alcohol group = 33.5 years). The wives of individuals with alcohol dependence had higher educational qualifications than the wives of individuals with drug dependence. Most of the wives of individuals with alcohol dependence were more employed than those of individuals with drug dependence (*X* ^2^ = 1.96, df = 1, *p* < 0.05)
14.	(Panaghi et al. 2016), Iran	To determine the moderating effect of personality traits on the relationship between living with an addicted man and codependency	Case‐control research 140 women (70 wives of addicted men and 70 wives of nonaddicted men)	Spann–Fischer codependency scale NEO five factor inventory (NEO‐FFI)	The codependency scores of addicted men's spouses were significantly higher than those of other women (55.7 vs. 51.0, *p* < 0.05)	Thirty‐eight of the women in this study (28.1%) had a college education, and 97 patients (71.9%) had a postgraduate diploma. Of all participants, 28.1% were under 30, 37% were between 30 and 40, and 34.8% were over 40 years of age. Fifty women (37%) were working, and 85 (63%) were housewives
15.	(Atintas et al. 2018), Ankara, Turkey	To compare marital adjustment, codependency, marital power, depression, anxiety, and stress in wives of both alcoholics and nonalcoholics	A correlational design 100 women, of whom 50 were wives of alcoholics and the remaining 50 were wives of nonalcoholics	Demographic information form Marital adjustment test Codependency assessment tool (CODAT) Depression anxiety–stress scale (DASS‐42) Couple power scale	Wives of alcoholics tended to be more codependent and had a lower self‐perception of marital power than did those in the comparison group	The mean age of the group composed of spouses of alcoholics was 42.74 years (SD = 10.70), and the mean duration of their marriage was 19.86 years (SD = 12.10). In the comparison group, the mean age of participants was 39.60 (SD = 8.25), and the mean duration of marriage was 13.62 years (SD = 9.63)
16.	(Noriega et al. 2008)	Determine the prevalence of codependence in young women who sought primary health care at a Mexico City Health Center, and (2) assess the contribution of selected risk factors for the probability of codependence	Descriptive, exploratory study 845 young women seeking primary health care in Mexico City	No prevalent rate is given		The descriptive analysis of the sample yielded the following: sociodemographic data: means of 31 years of age (SE 6.80), 10.15 (SE 6.44) years of marriage or living with one's partner, 2.13 (SE 1.12) children, and 34.42 (SE 8.39) age of spouse or partner. The educational attainment variable was associated with a lower probability of codependence (OR 544) and may operate as a protective factor
17.	(Padmapriya et al. 2025), India	Assess the existing level of depression and codependency among wives of alcoholics	Exploratory research design 100 wives of alcoholics who met the inclusion criteria were recruited using convenience sampling	Zung self‐rating depression scale Codependency scale	Study revealed that 96 (96%) of wives of alcoholics had moderate depression and 98 (98%) were moderately codependent, and 2% had highly codependent behaviors	Many of the wives of alcoholics, 38 (38%), were aged between 35 and 40 years and belonged to a nuclear family, had been married for 3–4 years, and were graduates. Most of them (38%) were housewives, 39 (39%) had a monthly family income of Rs. 10,000–Rs. 20,000
18.	(Padmavathi et al. 2014), India	To assess the codependency and depressive symptoms among wives of alcoholics	Descriptive research design 30 wives of alcoholics who fulfilled the inclusion criteria were selected by purposive sampling	Codependency scale Center for epidemiologic studies depression scale (CES‐D)	Many of the wives (90%) had high codependency, and 94% of them had mild depression	Most of the wives (36%) were in the age group of 41–50 years, Hindus (93%), and the majority (70%) from a joint family, belonged to conjugal marriage, and were living with their husband for above 10 years

A data extraction form was developed, including the following:

1. Publication details: Author(s), year, country, and study design.
2. Participant characteristics: Sample size, demographics, and alcohol use severity.
3. Findings: Prevalence, psychological/social/health impacts, associated factors, and risk factors.

#### 2.1.7 Stage 5: Collating, Summarizing, and Reporting the Results

The data were summarized narratively, supported by figures and tables. Information extracted from each included study comprised the authors’ names, year and journal of publication, study purpose, geographic location, sample size, study design, and measured outcomes. No risk of bias assessment or advanced statistical analysis was conducted, as this review aimed to provide an overview of existing evidence rather than evaluate study quality.

##### 2.1.7.1 Narrative Synthesis

The range of included studies was mapped, and key thematic areas were identified and summarized.

##### 2.1.7.2 Basic Qualitative Content Analysis

Evidence on associated factors was synthesized using this approach. Themes were inductively derived from the extracted data, resulting in the overarching categories and a conceptual framework describing factors associated with codependency.

#### 2.1.8 Stage 6: Consult With Stakeholders

To improve the clinical relevance of the findings and to validate the research questions and data extraction tool, input was obtained from mental health specialists to strengthen the validity and pertinence of the scoping review.

### 2.2 Gaps and Implications

We highlighted areas for further research and development of the intervention.

### 2.3 Registration

To ensure reproducibility and transparency, the review was registered with the Open Science Framework (OSF) (https://doi.org/10.17605/OSF.IO/4RS83).

## 3 Results

**FIGURE 1:**
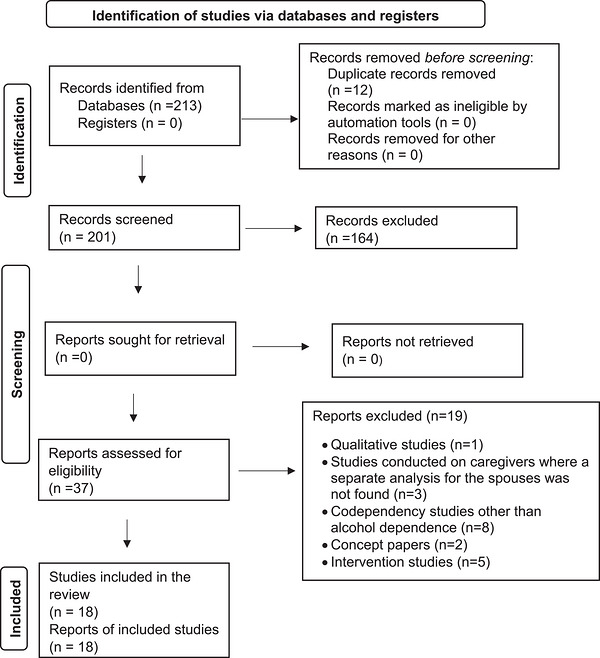
Flowchart illustrating the search and screening process.

This review synthesized evidence from eighteen observational studies involving approximately 2299 participants across four countries, India, Iran, Pakistan, and Turkey, providing a comprehensive understanding of codependency among wives of alcohol users. The geographical distribution of included studies from these countries was not the result of an intentional regional restriction but rather reflects the current availability of eligible studies meeting the predefined inclusion criteria. Of the 213 studies initially identified, 18 studies met the inclusion criteria and were included in the review after full‐text screening (Figure 1). All the included studies focused on wives of alcohol users who were living with their husbands.

### 3.1 Assessment of Codependency Among Wives of Alcohol Users

The included studies reported the prevalence rates of codependency among wives of alcohol users. Various instruments were employed to measure codependency, including the Span–Fisher Codependency Scale [4, 12, 34, 35, 36], the Codependency Assessment Scale [3, 5, 37, 38, 39], the Codependency Assessment Questionnaire [10, 40], and the Codependency Assessment Tool [41].

### 3.2 Reported Prevalence Rates of Codependency Among Wives of Alcohol Users

**FIGURE 2:**
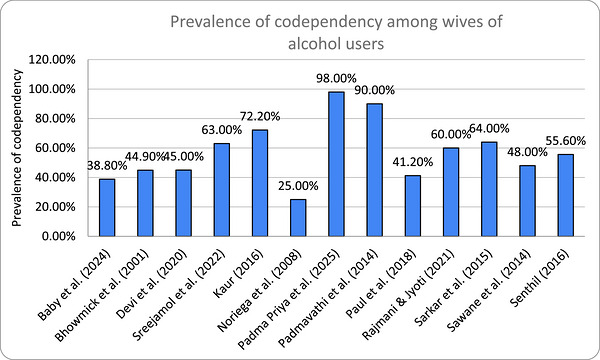
Prevalence of codependency among wives of alcohol users across included studies.

Among the 18 reviewed studies, 13 studies revealed varying levels of codependency among wives of alcohol users (Figure 2). Most studies reported moderate to severe levels of codependency. Severe codependency ranged from 41% to 92% [12, 34, 37], whereas moderate levels ranged from 34% to 98% [9, 39]. Several studies also identified significant differences between the spouses of alcoholics and nonalcoholics [3, 42]. Additionally, depression frequently co‐occurred, with 80% of participants exhibiting major depression and 20% mild to moderate depression [38]. In contrast, Baby et al. [42] reported a lower prevalence, with 57.67%1.% of wives showing low levels of codependency.

### 3.3 Factors Associated With Codependency

This scoping review examining the factors associated with codependency among spouses of individuals with AUDs highlights several interconnected domains. Psychological aspects emerged as the most frequently reported theme across studies. Numerous studies [4, 5, 6, 9, 34, 38, 39, 43] documented a high prevalence of depression and other psychological disorders in this population. Sarkar et al. [10] further emphasized psychological vulnerabilities such as fear and shame. They disrupted identity development, whereas Atintaş and Tutarel‐Kışlak [40] reported significant symptoms of stress, anxiety, and depression associated with marital power dynamics. Research by Paul et al. [12], Nandhini [37], Rajamani [36], and Zaidi [13] highlighted poor marital quality and reduced interpersonal satisfaction, identifying marital and relationship quality as another critical domain. Additionally, family environment factors, including unhealthy patterns of family interaction, were noted by Senthil [3].

The personality traits area included findings on neuroticism [41] and dependent, anxious, and borderline personality characteristics [42]. Social support and coping inadequacies were also reported [43]. Bhowmick et al. suggest that limited support networks and ineffective coping mechanisms exacerbate codependency [29]. Moreover, Noriega et al. [8] underscored the influence of trauma and abuse experiences, particularly physical and sexual mistreatment, in the development of codependency. Taken together, these studies illustrate a complex interplay of psychological distress, strained marital relations, maladaptive personality traits, dysfunctional family environments, poor coping resources, and experiences of trauma, all reinforcing the cycle of codependency among wives of alcohol users. These aspects are thematically categorized into three broad domains in the thematic map (Table 3): psychological, social, and physical.

**TABLE 3:** Thematic map of factors associated with codependency.

Domain	Factors associated with codependency	References
**Psychological**	Depressive symptoms	[5, 9, 34, 38, 39, 43]
	Higher levels of depression, anxiety, and stress symptoms	[40]
	Psychological issues	[6]
	Neuroticism	[41]
	Psychiatric comorbidity	[4]
	Fear, shame, and impaired identity development	[10]
	Dependent, anxious, and borderline personality traits	[42]
**Social**	Poor quality of marital life	[12, 36]
	Unhealthy family interaction patterns	[3]
	Poor interpersonal satisfaction	[13]
	Low coping resources and social support	[29]
	Lower self‐perception of marital power	[40]
**Physical**	Physical or sexual mistreatment	[8]

## 4 Discussion

The prevalence and extent of codependency among spouses of individuals with AUDs vary, as highlighted in the present scoping review. Many of these women exhibit moderate to severe levels of codependency. Notably, codependency is not confined to India; significant levels have also been documented in other countries among spouses of individuals with AUDs. For instance, Marks et al. [44] reported that in the United States, wives of men with alcohol dependence scored significantly higher on codependency inventories than wives of men without alcohol dependence, suggesting that marital dynamics associated with alcohol use are universally comparable. Similarly, a Turkish study by Atintaş and Tutarel‐Kışlak [40] found that wives of men with alcoholism experienced markedly higher levels of stress, anxiety, and depression, which were strongly correlated with increased codependency. This cross‐cultural consistency underscores that the psychological burden and relational challenges experienced by spouses of individuals with alcohol dependence transcend social and cultural boundaries.

Methodological variations, such as differences in codependency measurement tools, sampling methods, cultural expectations regarding marital roles, and the availability of social support, may account for the observed disparities in the severity of codependency across studies. For instance, Baby et al. [42] reported that 57.61% of wives exhibited low codependency scores, whereas Nandhini [37] found that up to 92% of wives were highly codependent. These contrasting findings suggest that protective factors, such as strong community and family support, may mitigate the negative psychological effects of living with an alcoholic partner. The psychological consequences of codependency are further evidenced by research linking it to anxiety, depression, and low self‐esteem. Devi et al. [38] found that the severity of depression was directly proportional to the degree of codependency. These findings are consistent with national and international research: Adhikari et al. [45] reported a positive correlation between codependency and symptoms of anxiety and depression among spouses of alcoholics admitted to a teaching hospital in Nepal. Zaidi identified a relationship between codependency and interpersonal satisfaction among Pakistani spouses of alcohol abusers [13], whereas Dear and Roberts [46] observed that codependent individuals frequently experience heightened psychological distress. The dynamics of relationships and family systems also play a crucial role in sustaining codependency. Rajamani [36] found that more than 60% of spouses exhibited moderate levels of codependent behaviors, possibly reflecting complex family roles and resilience factors. Similarly, Sarkar et al. [10] reported that 64% of wives displayed codependent behaviors characterized by fear, shame, and delayed identity development. Supporting this, international studies have demonstrated that maladaptive codependent patterns are often reinforced by inadequate family support systems [29, 47].

Across the included studies, the prevalence estimates summarized in Figure 2 indicate that codependency is widely reported among wives of alcohol users, although the magnitude varies considerably between studies. Despite the variability (25%–98%), most studies reported moderate to high levels of codependent behaviors, suggesting that psychological and relational strain is a common experience among spouses of individuals with alcohol use problems. This overall pattern highlights the importance of recognizing codependency as a significant psychosocial issue in families affected by alcohol use.

The wide variability in reported prevalence of codependency across the included studies, ranging from relatively low to extremely high estimates, can be largely attributed to methodological and contextual differences. First, there was significant variation in the instruments, score ranges, and cut‐off values utilized by various researchers to classify severity levels when evaluating codependency. The prevalence estimates’ direct comparability between studies is restricted by these discrepancies. Second, different study settings seem to have affected reported prevalence; higher levels of codependency are frequently seen in hospital‐based or de‐addiction center samples as opposed to community‐based studies, which probably reflects higher clinical severity and help‐seeking behavior. Third, certain studies may have included selection bias and exaggerated prevalence estimates due to variations in sampling tactics, such as the preponderance of non‐probability approaches like convenience or purposive sampling. Lastly, observed variability is further influenced by participant characteristics and sociocultural environment, including length of marriage, the intensity and duration of the partner's alcohol use, and economic reliance. Focusing on marital status may have increased the likelihood of missing relevant information when publications did not explicitly target wives but all forms of partnerships. This may have led to the exclusion of studies involving individuals in common‐law relationships or women who fulfill spousal roles without legal marital status. When combined, these elements emphasize the necessity of interpreting prevalence results with caution and stress the significance of context‐sensitive study methods and standardized measuring techniques in subsequent investigations. A synthesis of prevalence data cannot be justified methodologically since this is a scoping review.

Taken together, these findings underscore the significant emotional and interpersonal burden borne by spouses of individuals with AUDs. They emphasize the urgent need for comprehensive intervention strategies that address not only the needs of the individual with alcoholism but also those of family members, particularly spouses who often suffer in silence. Structured support systems, such as Al‐Anon groups, family therapy, and psychoeducational programs, can play a crucial role in alleviating codependency and its associated psychological consequences. Furthermore, future research should focus on identifying protective factors and developing culturally sensitive interventions that strengthen family resilience and help prevent severe codependency among spouses.

### 4.1 Strengths and Limitations of the Review

This review enhanced methodological transparency, rigor, and reproducibility by adhering to the JBI standards, the PRISMA‐ScR checklist, and the Arksey and O'Malley framework. Conceptual clarity was improved by the identification of key psychological, social, and physical domains using narrative synthesis and content analysis. Selective reporting was prevented, and transparency was increased by registering the review methodology with OSF.

As the study included English‐language papers, it is potentially missing evidence published in other languages. No formal risk‐of‐bias assessment was conducted in accordance with the scoping review criteria. This restricts the methodological robustness of the listed studies. Most studies included in this review employed cross‐sectional designs, which limit the ability to infer causal relationships between codependency and associated factors. Many studies were also based on relatively small sample sizes and conducted in single clinical or community settings, potentially restricting the representativeness of findings. The included studies were restricted to four countries, as the studies from other regions were either not available or did not meet the inclusion criteria, particularly with respect to reporting prevalence data among wives of individuals with AUD. Furthermore, non‐probability sampling methods, particularly convenience and purposive sampling, were commonly used, increasing the likelihood of selection bias and possibly inflating prevalence estimates. Variability in measurement tools and cut‐off scores further complicates comparisons across studies. Collectively, these methodological constraints suggest that while the evidence consistently highlights a high burden of codependency among wives of alcohol users, the findings should be interpreted with caution. Future research would benefit from larger, multi‐site studies employing standardized assessment tools and longitudinal designs to strengthen the evidence base.

## 5 Conclusion

This review highlights that codependency among wives of alcohol users is common, multifaceted, and shaped by psychological, social, and physical factors. The findings emphasize the need to view codependency not merely as an individual concern but within a broader family and societal framework. Integrating screening and targeted interventions into alcohol rehabilitation and community mental health programs can foster improved well‐being for both spouses and families. Future research should aim to deepen understanding through longitudinal and intervention‐based studies, with a focus on culturally responsive approaches that can effectively support these women and strengthen family systems.

## Author Contributions

**Binil Velayudhan** and **Savitha Prabhu** conceptualized the idea and design, searched, screened, extracted data, and wrote up the draft manuscript. **Prachi Pundir** has been involved in writing, reviewing and editing, supervision, and conceptualization. **Olga Gershuni** was involved in critically reviewing and revising the manuscript. All authors read and approved the final version.

## Funding

The authors have nothing to report.

## Disclosure

The source of the data was published literature.

## Ethics Statement

The authors have nothing to report.

## Conflicts of Interest

The authors declare no conflicts of interest.

## Data Availability

Data sharing not applicable to this article as no datasets were generated or analyzed during the current study.
